# Applying psycho-behavioural phenotyping in obesity characterization

**DOI:** 10.1007/s11154-023-09810-8

**Published:** 2023-06-01

**Authors:** Lucía Camacho-Barcia, Ignacio Lucas, Romina Miranda-Olivos, Susana Jiménez-Murcia, Fernando Fernández-Aranda

**Affiliations:** 1 Clinical Psychology Unit, University Hospital of Bellvitge, Barcelona, Spain; 2 Psychoneurobiology of Eating and Addictive Behaviours Group, Neurosciences Program, Bellvitge Biomedical Research Institute (IDIBELL), Barcelona, Spain; 3 CIBER Fisiopatología Obesidad y Nutrición (CIBERobn), Instituto de Salud Carlos III, Barcelona, Spain; 4 Department of Clinical Sciences, School of Medicine and Health Sciences, University of Barcelona, Barcelona, Spain

**Keywords:** Obesity, Phenotype, Psycho-behavioural, Reward dependence, Cognitive control, Emotional eating

## Abstract

Individual differences in obesity, beyond being explained by metabolic and medical complications, are understood by alterations in eating behaviour which underlie psychological processes. From this psychological perspective, studies have identified several potential characteristic features at the psycho-behavioural level that could additionally explain the maintenance of chronic excess weight or the unsuccessful results of current treatments. To date, despite the growing evidence, the heterogeneity of the psychological evidence associated with obesity has made it challenging to generate consensus on whether these psycho-behavioural phenotypes can be a complement to improve outcomes of existing interventions. For this reason, this narrative review is an overview focused on summarizing studies describing the psycho-behavioural phenotypes associated with obesity. Based on the literature, three psychological constructs have emerged: reward dependence, cognitive control, and mood and emotion. We discuss the clinical implications of stratifying and identifying these psycho-behavioural profiles as potential target for interventions which may ensure a better response to treatment in individuals with obesity. Our conclusions pointed out a considerable overlap between these psycho-behavioural phenotypes suggesting bidirectional interactions between them. These findings endorse the complexity of the psycho-behavioural features associated with obesity and reinforce the need to consider them in order to improve treatment outcomes.

## Introduction

Obesity is defined by the world health organisation (WHO) as an excessive accumulation of fat tissue, that stages a risk to health [1]. It is a chronic, complex, and multifactorial disease influenced by several factors including genetic, physiological, metabolic, psychological, social, and cultural ones. Due to the complexity of these interactions, weight management remains a major challenge in obesity treatments. This reaffirms the necessity to identify differences among persons with this pathology in order to enable specific treatment strategies [2]. In addition to the consequences in health derived from excessive adipose tissue accumulation, in some individuals, obesity can imply comorbid psychological problems [3]. For this reason, it is highly probable that these individuals would require psychological intervention, in parallel to traditional medical treatment. Furthermore, beyond the psychological discomfort directly associated with obesity, a small proportion will develop lifetime psychiatric disorders such as bipolar disorder, major depression disorder, attention deficit hyperactivity disorder (ADHD), or binge eating disorder (BED) [4].

The psychological study of obesity has gained interest during the last years because it has allowed defining its role in the development, maintenance, and prognosis of this disease. Beyond metabolic homeostasis, dysfunctions in cognitive, emotional, and motivational processing may characterise psycho-behavioural phenotypes which can underlie problematic behaviours related to obesity that might affect food intake and hinder the maintenance of normal weight, even after obesity interventions [5]. These problematic behavioural patterns related to dysfunctional eating in obesity may be characterised by a persistent and excessive consumption of energy-dense food [6] motivated by higher sensitivity to food reward [7], underlying problems in self-control over intake [8] or emotional states [9]. It should be noted that these problematic patterns are not independent of each other, but can overlap, increasing the complexity and heterogeneity of obesity. Nonetheless, interventions considering these psycho-behavioural factors in obesity can allow the implementation of individual-specific psychological strategies to ensure and sustain weight loss [8, 10].

Even though the identification of the psycho-behavioural mechanisms in obesity has been beneficial in clinical practice, to date, due to the heterogeneity of obesity it has not been possible to create a consensus to stratify and identify potential target interventions that guarantee a better response to treatment [11, 12]. One possible approach is to consider brain circuits linked to the psychological processes that underlie these behavioural phenotypes. These are mainly mesolimbic dopamine circuitry associated with reward sensitivity, prefrontal cortex regions related to dietary self-control, and the hypothalamic–pituitary–adrenal axis along with limbic system regions of the amygdala and hippocampus related to mood and stress reactivity. Phenotyping patients centred on these characteristics could help to create tools for health providers that allow them to offer more personalized care, and a more efficient weight management therapy.

This narrative review aimed to summarise the evidence related to studies focused on identifying and describing psycho-behavioural phenotypes in obesity, under an umbrella of three main underpinnings widely addressed in the psychoneurobiology fields: reward dependence, cognitive control, and mood and emotion. A better understanding of these psycho-behavioural mechanisms underlying obesity could be beneficial in developing individualized treatments or complementing the effectiveness of existing interventions.

## Reward dependence

When studying the psycho-behavioural phenotypes in obesity, one of the key aspects is the presence of reward sensitivity (RS) [13, 14]. The RS is a personality trait defined by approach and appetite responses toward stimuli or events perceived as rewarding [15]. In the context of food consumption, studies have described a high RS associated with a preference for unhealthy foods [16, 17] and overeating [18, 19] in patients with BED [7] and obesity [18, 19]. The tendency to prefer high-fat and high-sugar foods has emerged as a risk for obesity and high weight maintenance factor for some individuals with obesity [18, 20]. In this regard, some studies have investigated whether alterations in the motivational and reward processing underlie neuroendocrine mechanisms in an attempt to decipher maladaptive eating behaviour in obesity.

From a neurobiological perspective, it is hypothesized that the persistent and excessive consumption of high-sugar and fatty foods might have an impact on neuroendocrine and hedonic mechanisms that regulate hunger, satiety, and the rewarding aspects of food [21, 22]. For instance, insulin resistance precedes the development of type 2 diabetes, a common denominator in metabolic syndrome. Obesity is a risk factor for developing insulin resistance [23, 24]. High insulin levels are a response to elevated blood glucose levels. Chronically altered insulin levels are associated with the persistent consumption of high simple sugary foods, with high glycaemic index, meantime this insulin resistance could underlie the tendency for caloric foods [22, 25] and overeating [24]. A study showed that hyperinsulinemia positively correlated with the hyperactivation of limbic-striatal regions and increased sensitivity to highly rewarding food stimuli [25]. An experimental intranasal insulin application study showed that this administration route affects brain regions involved in inhibitory control and hypothalamic function if individuals presented higher levels of visceral fat [24]. These findings suggest that higher insulin levels are altering the processing of reward value and contributing to overeating.

On the other hand, studies have reported that altered circulating concentrations of ghrelin observed in obesity [26] could underlie alterations in food-related reward processing. Ghrelin concentrations tend to increase before each meal but decrease immediately after food intake [27]. However, in diet-induced obesity models of mice, it has been demonstrated that palatable diets might alter ghrelin signalling promoting obesogenic eating patterns [28, 29]. Consistent with this, results from a longitudinal study in humans that evaluated changes in endocrine factors (i.e., ghrelin, insulin, and leptin) in obesity assessed before and after bariatric surgery [30], reported a decrease in ghrelin, insulin, and leptin after the intervention. Additionally, they found that reduced ghrelin concentrations were positively associated with a reduced activation in the dorsolateral prefrontal cortex and a reduction in the craving ratio for high-calorie foods [30]. These results may indicate that, given its interaction with brain regions involved in self-control, ghrelin could regulate the preference for hypercaloric foods.

Reward hypersensitivity was originally associated with maladaptive behaviours observed in individuals with addictive patterns and substance use disorders [31–33]. In these individuals, there is an overvaluation of a reinforcing stimulus that overcomes self-control, ignoring potential future consequences or losing control of the rewarding stimulus. Under this theoretical model, some studies have discussed the existence of similar behavioural and neurobiological correlates in individuals with addictions and obesity, emerging the construct of Food Addiction (FA) [32, 34–36]. This model suggests that similar to addictive substances, in obesity, the consumption of a certain type of food can produce neuroadaptation in reward-related mechanism modulated by dopaminergic (DA) neurotransmission [36], an analogous phenomenon observed in addiction neurobiological pathways [14, 37–39]. Excessive consumption of high-sugar and high-fat foods can engage in desensitizing brain reward pathways displaying a downregulated dopaminergic system (i.e., reduced availability of DA receptors) in animals [14, 39] and individuals with obesity [38, 40]. Although not all subjects with obesity present FA, some psychological features described in FA, such as the intense desire to consume certain foods (i.e., craving), are highly reported [41–43]. Craving represents a risk factor for overeating because it anticipates the idea of food rewards increasing the expectancy for a future food intake [44]. Under this craving, some authors have characterised the eating behaviour of some individuals with obesity who have difficulties in self-controlling the craving for highly palatable foods [45, 46]. In these cases, food is perceived as highly rewarding and individuals constantly crave it, even without food cues or hunger sensations [43, 44].

Among the psychopathologies that can derive from these alterations in reward value over food stimulus are bulimia nervosa (BN) and BED. Both BN and BED are characterised by excessive overeating in short periods of time, usually of highly palatable food [47]. The coexistence of obesity and eating disorders could hinder recovery prognosis, since, in addition to the psychopathology, those individuals have greater difficulty in controlling eating when desired [43, 48, 49]. Studies evaluating reward-based decision-making in individuals with obesity with and without BED have reported mixed results [50–53]. Individuals with obesity tend to prefer an immediate but small reward, to a delayed reward compared to normal-weight controls [51], but the results diverge depending on the rewarding stimulus used [54]. If the reward stimulus is food, most studies report a greater preference for immediate rewards in individuals with obesity compared to normal-weight controls [51], but these differences do not occur as often if the reward stimulus is money [53, 55]. Therefore, the food stimulus plays a significant role in the description of a psycho-behavioural phenotype based on reward dependence.

### Clinical implications

From a clinical perspective, a higher neurobehavioral sensitivity to reward towards high-calorie food can diminish success for weight loss in obesity [56, 57]. Consequently, in recent years, some ongoing research aimed to identify interventions that train and reduce hypersensitivity, as well as correct the attentional bias toward high-calorie food stimuli [54, 58–60]. These interventions have been preliminarily demonstrated to have a positive impact on reducing food craving [54, 60], overeating [59], and weight gain [58]. Future interventional studies should work to investigate how psychological intervention addressed to improve motivational drivers towards food and inhibitory control training can benefit the specific neurocognitive processes involved in the recovery of normal weight in obesity [7].

## Cognitive control

Cognitive control is a multi-dimensional construct that refers to the ability to coordinate our behaviours [61, 62] considering the context and individual goals, countering automatic impulses [63]. This control involves either the inhibitory control of automatic responses, changing the attentional focus or regulating emotional responses [64]. All these cognitive control processes can regulate eating-related behaviours, by modulating the automatic responses to feeding-associated stimuli (e.g. high caloric palatable food or hunger signalling) [65]. Numerous studies have addressed inhibitory control in obesity under the hypothesis that problems in the regulation of eating behaviour may be related to difficulties in self-control over food intake [8, 19, 50, 66]. Deficits in inhibitory control have been associated with impulsive behaviours in obesity [67] and increased body mass index (BMI) [68–70], suggesting that difficulties with self-control might underlie the risk of overeating in some individuals. Indeed, it has been observed that the combination of deficits in inhibitory control along with alterations in reward processing, may increase the risk of excessive food consumption in the absence of metabolic needs [71]. Especially when individuals with obesity are faced with high-calorie foods, greater difficulty in restraining their impulses to eat is observed [65, 72]. Likewise, these difficulties in controlling approach behaviour towards food cues, as well as in stopping intake when the endocrine-mediated satiety signal informs of reaching energy balance [21, 72, 73] have predicted weight gain and its maintenance [19, 56, 57, 74–77].

As a general rule, the presence of impulsivity has been related to a lack of inhibitory control representing a risk factor for obesity [78]. Although there is not a wide consensus for identifying impulsivity as a valid psycho-behavioural phenotype in obesity [76], some studies have highlighted that impulsive behaviour is frequently observed in these individuals, especially if stimuli are food displaying an inability to delay gratifications [53, 79]. Problems to delay gratification are understood as a preference for immediate but smaller rewards over delayed but larger rewards. In obesity, this bias toward immediate rewards is associated with the preference to immediately consume high-calorie food, disregarding future health consequences. This inability to delay gratification can predict diminished success with weight loss in obesity treatment even in children and adolescents [80]. Recent studies prospectively reported that childhood deficits in self-control can determine future physical health and predict the development of obesity at early ages [81, 82]. Furthermore, in addition to decision-making based on rewards, individuals with obesity tend to also exhibit riskier decision-making compared to normal weight control [21, 83, 84]. Under ambiguity, individuals with obesity showed a tendency to risky decisions-make compared to control, emerging the question of whether this type of decision can imply consequences in daily life or in relation to the weight loss treatment [83, 84].

From a neurobiological point of view, cognitive control is orchestrated by the prefrontal cortex [63]. The prefrontal cortex plays a prominent role in the regulation and modulation of eating behaviour [73], in addition to its involvement in attentional, motivational, planning, and decision-making processes [85]. Its role in emotional regulation is explained by its connection with limbic structures such as the amygdala, hypothalamus, and insula [86, 87]. Volkow et al. [88] observed a negative correlation between BMI and prefrontal metabolic activity linked to impaired performance of individuals with obesity in neurocognitive tests. in experimental conditions analogous to everyday food choices. In this line, another study reported that the activity of the dorsolateral prefrontal cortex in a delay discounting task was linked with weight maintenance in patients with obesity after a dietary treatment [54]. This area has been specifically associated with impulse control mechanisms [80]. A dysfunctional activation in the prefrontal cortex has been associated with deficits in attentional processes and behavioural planning in individuals with obesity, who can engage in impulse behaviours without considering the potentially hazardous health consequences [19, 72, 89, 90].

Overall, the overconsumption of high-calorie foods can impact the interaction between prefrontal regions and endocrine signalling, such as leptin [91, 92] and insulin [24]. Both are anorexigenic hormones involved in reducing intake through satiety signals. Studies have demonstrated that insulin and leptin resistance observed in individuals with obesity can alter the prefrontal function as well as the cognitive function that regulates food intake. In that regard, a narrative review suggested that leptin might be a therapeutic target potentiating prefrontal activity when individuals with obesity are exposed to food [21].

From psychiatric epidemiology, it has been identified that there is a comorbidity between psychopathologies related to impulse control and attentional problems and obesity. Thus, studies report that obesity may present with attention deficit hyperactivity disorder [93], high comorbidity with substance abuse disorder [94], and binge spectrum disorders [95–97]. All these psychological disorders present a higher prevalence in individuals with obesity than in the general population with normal weight [98–100].

In addition to the straightforward relationship between lack of cognitive control and obesity, the literature also points out a potential association with inflammatory processes linked to overconsumption of food [101–103]. In the context an impaired cognitive functioning in obesity, studies have observed that inflammatory processes could underlie impulse behavioural patterns [101–103] which can also collaborate in maintaining dysfunctional obesogenic patterns and leading to a high-severe enduring condition with difficult treatment [74, 103].

### Clinical implications

Impulsivity is a multi-faceted construct [104] whose features represent risk factors for obesity [67] and have been associated with unsuccessful outcomes of its treatment [74, 75, 77]. However, prefrontal cognitive control could also serve as a potential target for the treatment of obesity [105]. From a psycho-behavioural perspective, interventions designed for specific disorders, like ADHD or BED -characterized by high impulsive behaviours and lack of control- could also be useful for patients with obesity, even for those that do not necessarily present one of these psychological disorders. In this sense, strengthening conscientiousness and self-control skills could be potential strategies in order to effectively treat obesity from the lack of cognitive control [106, 107]. Moreover, non-invasive deep magnetic brain stimulation is another treatment approach that has shown promising results in the reduction of impulse-related behaviours associated with obesity [108]. Problems in attention and consciousness can also be improved in obesity by mindfulness-based interventions. Mindfulness has already been reported good outcomes for the treatment of obesity, producing changes in eating behaviours [109].

## Mood and emotion

### Mood and obesity

Psychological disturbances that act on the persons’ mood are highly prevalent in individuals with obesity [110]. Different studies acknowledging numerous differential variables, such as age, sex, and race have found high associations between obesity and depressive and anxiety symptomatology [3], bipolar disorder [111], premenstrual syndrome in women and seasonal affective disorder [112]. At the same time, this relationship seems to be bidirectional, as the presence of obesity may increase the risk of developing these types of disorders [110, 113].

There is a big iatrogenic role played by psychiatric medications used to treat some of these disorders that promote weight gain, affecting both appetite and sedentarism behaviours [114]. Independently of the indicated pharmacotherapy, some of these disorders' characteristic symptoms, such as sleep disturbances, disordered eating, disproportionated need for food, and sedentarism behaviour may increase the risk of weight-related problems [115]. Likewise, other affective and lifestyle associated factors, like low self-esteem and social isolation may drive these patients to consume large quantities of foods that provide feelings of well-being, consolation and comfort [112], most likely highly palatable and caloric foods, with high content of fats and sugars [116].

Additionally, some authors have recognised that higher levels of emotional dysregulation are strongly associated with increased depression and anxiety symptomatology [9, 117]; which may rise the risk of emotional eating and later obesity. Results from a longitudinal study showed that emotional eating mediated a positive association between depression and an increment in BMI and waist circumference over 7 years of follow-up. Furthermore, they found that those individuals with higher emotional eating and shorter duration of sleep were especially susceptible to weight gain [118].

There are several possible neurobiological associations between these disorders. One of these is the effect of obesity on the brain’s production and release of serotonin. This neurotransmitter signalling, highly engaged in both mood and satiety pathways [119], seems to be decreased in the presence of obesity [120]. At the same time, diets with high content of fats, highly consumed as comfort foods in these types of patients, can meddle with serotonin production leading to changes in the mood [121].

Another proposed common connection is the increment in cortisol levels, observed both in depression symptoms and the risk of obesity. The increase in this hormone produces changes on the hypothalamic–pituitary–adrenal axis, generating a predisposition of visceral adipose tissue gain and deposition [122]. As well, sleep disturbances and subsequent disruptions in the circadian rhythm could also be affecting both leptin and ghrelin secretion, fundamental hormones in the satiety-appetite cascade contributing to disturbances in the control of food intake [23]. Chronic inflammation may also be mediating this association. Mood disorders and obesity have been linked to elevated levels of several inflammatory markers [123, 124].

#### Clinical implications

It is important to acknowledge that for patients with obesity, weight management is very complicated to achieve. As mood disturbances enter the equation and engage in a bidirectional relationship, interventions become more difficult to implement, and harder to achieve positive results. This population is at elevated risk of weight gain, in need of target interventions, and weight management strategies that consider all different variables that are affecting their health, both weight gain and psychological distress. Acknowledging the individualities of the mediating effects of this bidirectional relationship is fundamental. Evidence shows that patients with obesity achieved a significant reduction of body weight after engaging in personalized cognitive behavioural therapy for obesity. Moreover, those who reduced their body weight had a significant improvement in obesity-related quality of life, as well as a significative reduction in psychopathological symptomatology of depression and anxiety, and in cardiovascular risk factors [125]. As well, some specific nutritional interventions have been proposed as evidence of the impact of diet and nutrition on mental health. Incorporating a healthy dietary pattern, such as the Mediterranean diet, has exhibited reductions in the risk of mood disorders, meanwhile, modulating chronic inflammation and regulating body weight in the long term [126–128].

### Emotions, eating behaviour and obesity

Emotions play a significant role in eating behaviour, affecting food selection, and interfering with both quality and quantity of food consumption. Food can provide feelings of well-being and emotional comfort, but for some people, it can become a maladaptive emotional regulation strategy [118, 129]. Emotional eating (EE) can be defined as a tendency to eat in order to cope with negative emotions and stress [9]. It is a problematic eating behaviour, where individuals consume large quantities of “comfort food” in order to manage emotional states. As these foods tend to be high in sugars and fat, as well as in caloric content, engaging in EE could lead to an increase in body weight [130–132], and has been proposed as a major risk factor for obesity [133]. Concurrently, individuals with overweight or obesity have shown less effective negative emotional management, leading to EE [134]. Several people with obesity describe consuming foods, especially high-calorie and palatable foods, in response to emotional states [118]. Some authors indicate that the most frequent negative emotions affecting eating responses and increasing food intake would be anxiety, apathy, frustration, stress [135], loneliness [136] and boredom proneness [137, 138].

Emotions affect the eating response in several phases of the ingestion process: choice, motivation to eat, affective responses to food, and speed of eating [139]. It has been suggested that for some individuals, strong emotional cues may cause insensitivity to hunger and satiety signals, making them rely on external factors to initiate and stop eating [137, 140]. Some results have shown that individuals that experienced loss of control over eating, and consequently insensitivity to internal cues of hunger and satiety, reported higher levels of EE [141]. Findings from a recent systematic review showed that EE was significantly and positively associated with reward sensitivity [13]. Various authors have proposed that individual differences in sensitivity to reward stimuli, like highly palatable foods, may promote the overconsumption of these types of foods, increasing the risk of obesity [6]. Comfort food could be a source of reinforcement, and individuals with high reward sensitivity could engage in disproportional eating of these types of foods [18]. In the same line, Volkow et al. studied the association of dopamine with eating behaviours, including emotional eating. Their results showed that emotionality was negatively correlated with dopamine receptors in the dorsal striatum, suggesting that dopamine is involved with emotional components regulating eating behaviours [142].

Additionally, stress may be playing a critical role in food selection, promoting the consumption of 'comfort' foods in individuals with overweight or obesity [143, 144]. Stress can induce glucocorticoid release that may trigger increased consumption of high-sugary foods and insulin levels in consequence, promoting obesogenic physiological mechanisms [145, 146]. In fact, it has been observed that certain individuals with obesity, compared to normal-weight individuals, tend to change their dietary patterns by choosing highly palatable foods, rich in sugars and fats when they reported high perceived stress whereas normal-weight individuals tend to show dietary restraint [143–145]. This increased consumption of certain types of food has been associated with dysfunctions in the interaction between the HPA axis and limbic brain circuitry [147]. Both are interconnected through the hypothalamus and can be dysregulated by stress or emotional states, overriding energy balance, and becoming a vulnerability factor for an obesity phenotype [145]. The connection between the HPA axis and the limbic system is the amygdala in communication with the insula and the anterior cingulate cortex modulates the emotional response depending on the individual's interoceptive information and motivation towards this emotional context [87]. Alterations between the limbic system and the prefrontal cortex have been associated with greater difficulties in emotional regulation [148].

#### Clinical implications

Evidence indicates that those individuals with higher susceptibility to EE would benefit from interventions that allow them to develop emotion regulation skills, including psychotherapeutic interventions designed to reduce emotion dysregulation [9]. It is important to acknowledge that individuals with obesity may present difficulties identifying everyday emotions from those related to feelings of appetite and satiety. Thus, it is essential for them to cultivate abilities to recognize them.

Different tools have been proposed in order to help them to manage their emotions, such as the development of their abilities to redirect attention, and the management of the physiological consequences of the emotion [149]. Some authors have proposed interventions with dialectical behaviour therapy [150], as other interventions incorporated mindfulness [151] training may be in form of mindful eating, as a viable strategy.

## Concluding remarks

**Fig. 1:**
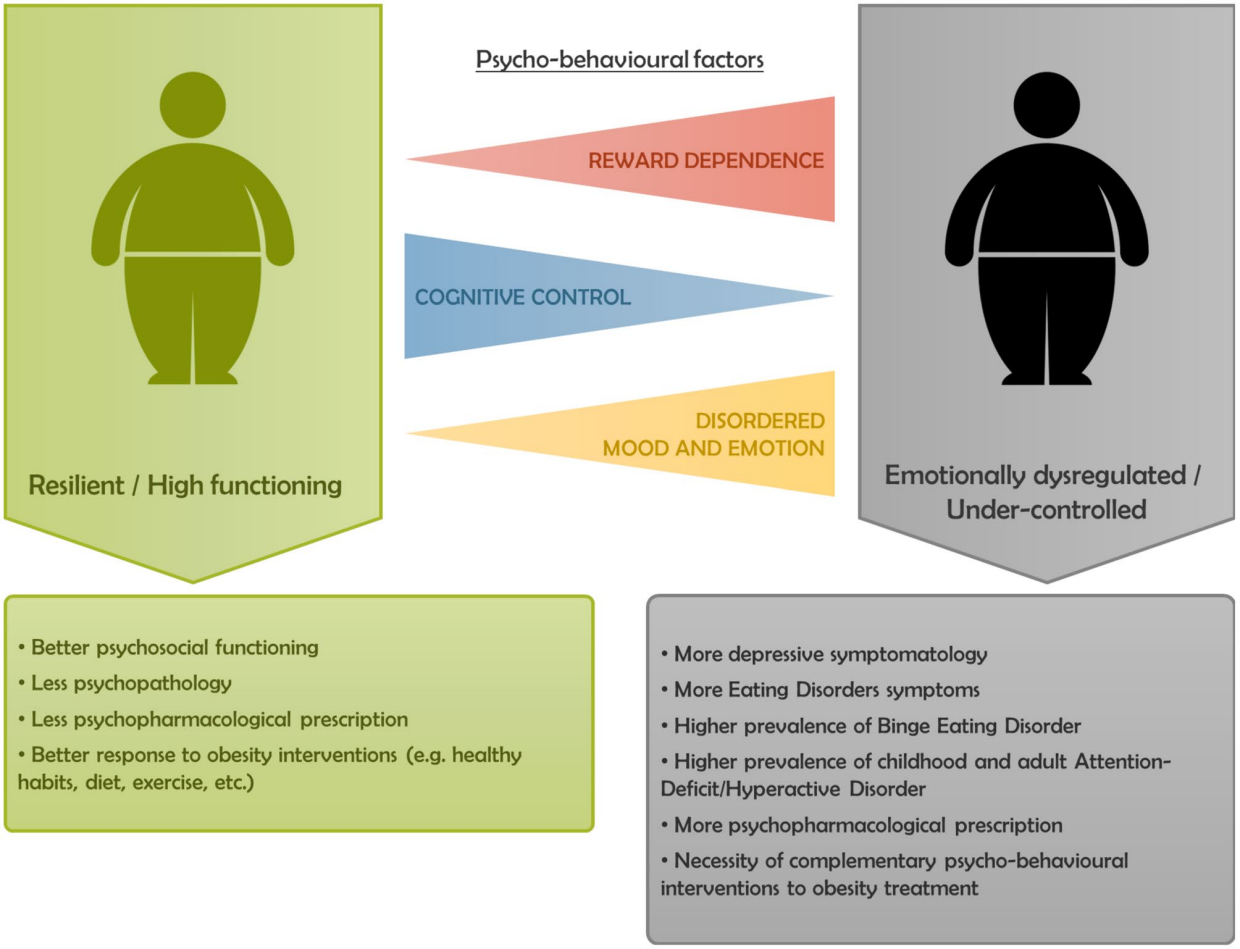
Differences between the resilient/high-functioning cluster versus the emotionally dysregulated/under-controlled cluster

In this review, we compiled evidence on psycho-behavioural phenotypes in individuals with obesity supporting the idea of heterogeneity in this condition. Previous research has identified two different clusters of patients with obesity: a resilient/high-functioning cluster and an emotionally dysregulated/under-controlled cluster [5]. Individuals in the functional cluster may have a more "metabolic" type of obesity, where weight gain susceptibility is associated with metabolic factors such as low basal metabolic rate or low capacity for fat oxidation [152]. This phenotype in obesity can be benefited from incorporating healthy habits, like adequate nutrition and physical activity. On the other hand, individuals in the dysfunctional cluster are more likely to have dysregulated behavioural patterns, develop mental disorders such as BED or ADHD, or experience depressive symptoms (Fig. 1). The present review was focused on this dysfunctional cluster, where the introduction of an additional psycho-behavioural treatment would be beneficial to achieve better outcomes in these individuals with obesity.

**Fig. 2:**
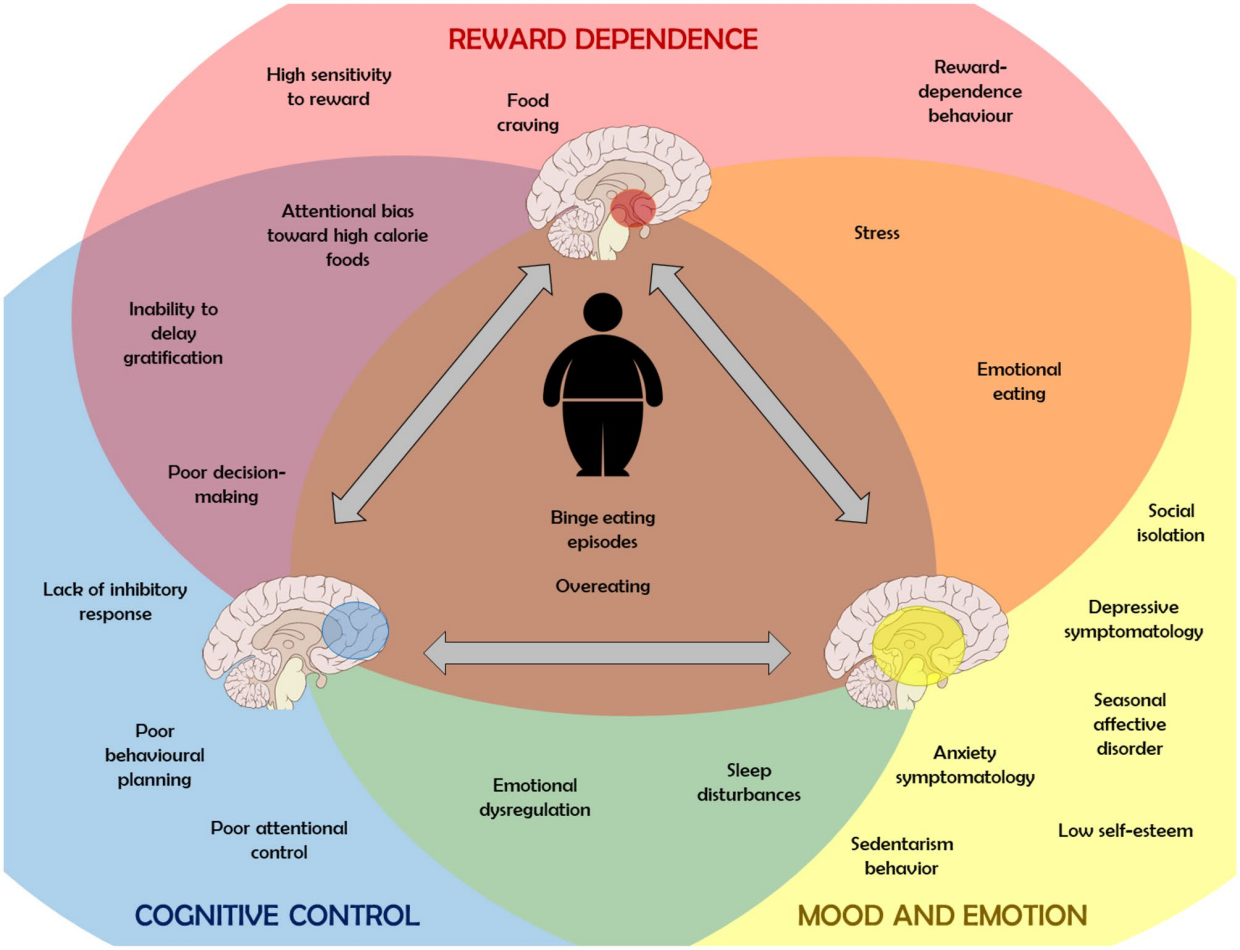
Applying psycho-behavioural phenotyping in obesity

We focused on three principal psychoneurobiology-related fields in this dysfunctional cluster: reward dependence, cognitive control, and mood and emotion. Figure 2 shows the different characteristic psycho-behaviours of each phenotype that has been directly related to obesity. Although some of these characteristics are able to differentiate between obesity phenotypes, it is interesting to recognize the existence of overlaps and bidirectional interactions between them which ratifies the complexity underlying this clinical population. Likewise, although these behavioural overlaps could complicate the differentiation between phenotypes, this review allows us to conclude the relevance that the identification of some of these psycho-behavioural patterns could have in clinical practice. A more predominant dysfunctional process could help to establish a more personalized therapeutic approach considering the current difficulties of some patients associated with weight loss and the control of metabolic disorders in obesity. Table 1 summarizes the main interventions to date developed to address these problems.

**Table 1:** Principal interventions to date developed to address the problems of presenting in psycho-behavioural phenotypes in obesity

Authors (year)	Intervention	Description	Aims	Dose	Results
Brockmeyer et al. (2015) [60]	Cognitive bias modification program: Food Approach–Avoidance Task (Food AAT)	Images of food and other objects on a computer screen that, with a joystick, they must zoom in or out depending on the images	To investigate the effects of approach and avoidance behaviour on food choice and consumption.	5 weeks (sessions of 15 minutes, twice weekly sessions)	- Reduction in attentional bias towards high-calorie food
					- Reduction food craving trait and cue-elicited food craving
					- Reduction in ED symptomatology
Kakoschke et al. (2018) [54]	A smartphone app named: Tilt Task (AAT + EFT)	AAT: an approach-avoidance training EFT: episodic future thinking	To compare AAT and EFT trainings. AAT: To reduce attentional bias toward high-calorie food EFT: health goals to be achieved in the next 4 weeks	1 week (AAT) and 4 weeks (EFT)	- Both trainings were motivating and easy to use.
					- AAT reduced approach bias for unhealthy food, increased healthy food choices, and reduced weight
					- EFT: no effect
O'Neill et al. (2016) [59]	EFT in natural environment	Participants had many food options at four different food court outlets 5 future health goals they would like to achieve in about 3 weeks	To modify eating habits in natural eating environments	1h of dinner session (participants' usual dinner time)	- Reduction in consumption of total and percent calories, fat, and an increase in percent calories from protein
					- Improvement in consumption of proteins
Luzi et al. (2021) [108]	deep Transcranial Magnetic Stimulation (dTMS)	dTMS was used to stimulate the prefrontal cortex (PFC) and insula	To investigate the effect of dTMS on anxiety, depression, impulsivity, foodcraving levels	5 weeks (3 times per week; ~30 minutes) = 15 treatment sessions	- Significant decrease in body weight and BMI
					- Reduction of impulsivity levels
Vermeiren et al. (2021) [107]	Multidisciplinary obesitytreatment (MOT) and Self-Control Training program	Computer-based training, attention training and behavioural inhibition training. Intervention in dietary patterns and physical activity with cognitive behavioural therapy and parental involvement	to assess the efficacy of short- and long-term an internet-based self-control programs focused on inhibition and attentional bias in an inpatient or outpatient children with obesity	6 weeks (2 sessions a week; 12 sessions)	- Reduction in BMI
					- Number of sessions was more beneficial in the self-control of the youngest study participants.

From the patient perspective, research has shown that individuals with obesity or other chronic metabolic disorders, such as diabetes, and mental health problems often do not seek help for psychological distress and do not receive adequate care [153]. Individuals with obesity usually expressed a desire for a more personalized and person-centred approach to identify and address the interconnected issues of living with co-existing metabolic disorders, psychological and behavioural problems, and illnesses such as diabetes [154]. Therefore, a better understanding of the psycho-behavioural mechanisms underlying obesity and other metabolic disorders could aid in the development of individualized treatment strategies complementing the effectiveness of existing interventions.

## Abbreviations

ADHD: Attention deficit hyperactivity disorder
BED: Binge eating disorder
BN: Bulimia nervosa
BMI: Body mass index
DA: Dopamine/dopaminergic
EE: Emotional eating
FA: Food Addiction
RS: Reward sensitivity
